# Prediction Score for Antimony Treatment Failure in Patients with Ulcerative Leishmaniasis Lesions

**DOI:** 10.1371/journal.pntd.0001656

**Published:** 2012-06-12

**Authors:** Cristian Valencia, Jorge Arévalo, Jean Claude Dujardin, Alejandro Llanos-Cuentas, François Chappuis, Mirko Zimic

**Affiliations:** 1 Instituto de Medicina Tropical ″Alexander von Humboldt,″ Universidad Peruana Cayetano Heredia (UPCH), Lima, Peru; 2 Laboratorios de Investigación y Desarrollo, Facultad de Ciencias y Filosofía, Universidad Peruana Cayetano Heredia, Lima, Peru; 3 Institute of Tropical Medicine Antwerp, Antwerp, Belgium; 4 Hospitaux Universitaires de Geneve, Geneve, Switzerland; 5 Unidad de Bioinformática, Laboratorios de Investigación y Desarrollo, Facultad de Ciencias y Filosofía, Universidad Peruana Cayetano Heredia, Lima, Peru; Hospital Universitário, Brazil

## Abstract

**Background:**

Increased rates for failure in leishmaniasis antimony treatment have been recently recognized worldwide. Although several risk factors have been identified there is no clinical score to predict antimony therapy failure of cutaneous leishmaniasis.

**Methods:**

A case control study was conducted in Peru from 2001 to 2004. 171 patients were treated with pentavalent antimony and followed up to at least 6 months to determine cure or failure. Only patients with ulcerative cutaneous leishmaniasis (N = 87) were considered for data analysis. Epidemiological, demographical, clinical and laboratory data were analyzed to identify risk factors for treatment failure. Two prognostic scores for antimonial treatment failure were tested for sensitivity and specificity to predict antimony therapy failure by comparison with treatment outcome.

**Results:**

Among 87 antimony-treated patients, 18 (21%) failed the treatment and 69 (79%) were cured. A novel risk factor for treatment failure was identified: presence of concomitant distant lesions. Patients presenting concomitant-distant lesions showed a 30.5-fold increase in the risk of treatment failure compared to other patients. The best prognostic score for antimonial treatment failure showed a sensitivity of 77.78% and specificity of 95.52% to predict antimony therapy failure.

**Conclusions:**

A prognostic score including a novel risk factor was able to predict antimonial treatment failure in cutaneous leishmaniasis with high specificity and sensitivity. This prognostic score presents practical advantages as it relies on clinical and epidemiological characteristics, easily obtained by physicians or health workers, and makes it a promising clinical tool that needs to be validated before their use for developing countries.

## Author Summary

The manuscript is relevant because of the finding of a new risk factor for chemotherapy failure and the development of a prognosis score for cutaneous leishmaniasis. The proportion of patients that have multiple lesions in American Tegumentary Leishmaniasis (ATL) is considerable. Publications and our experience permit to estimate that they represent around 20% of the affected population from the Amazon basin with cutaneous lesions. In addition, about 1/3 of them would correspond to the concomitant distant lesions category, the novel risk factor identified with a very high odds ratio (20–30) associated. Such numbers merit study of concomitant distant ulcers category on its own, not only because of clinical management implications, but also to search for factors that are contributing to chemotherapy failure. Finally, the simple equation proposed in the manuscript can be easily adapted to smart phone technologies. Similar prognosis equations are scarce for other pathologies and do not exist for Cutaneous Leishmaniasis at all. The simplicity of this tool should be followed by subsequent epidemiologic studies in other ATL endemic regions.

## Introduction

Leishmaniasis is caused by protozoan parasites of the genus *Leishmania* sp. There is an estimated 2 million new cases and almost 70 000 attributable deaths worldwide every year [1]. Leishmaniasis includes a cluster of diseases with widely diverse clinical manifestations. These include three major groups of clinical disorders: visceral leishmaniasis (VL), cutaneous leishmaniasis (CL), and mucocutaneous leishmaniasis (MCL) [2]–[4]. New world cutaneous Leishmaniasis (also known as American Tegumentary Leishmaniasis) is endemic in the Andean region affecting the most deprived socioeconomic groups.

Pentavalent antimonials (SbV) have been the first line drugs for leishmaniasis treatment for more than 50 years. Studies conducted in Latin America have reported rates of antimony failure ranging from 7% up to 39% [5]–[7], raising a serious concern on health policy makers. Several factors like the parasite species [8], [9], the duration of the period living in the endemic area [10], the duration of the presence of skin lesions before the start of treatment, and the presence of multiple cutaneous lesions, were found to be significantly associated with SbV therapy failure [9], [10].

The use of clinical scores as predictive tools is being more frequently used in several conditions. It provides a valuable tool for clinical management, orienting physicians to establish the most appropriate treatments [11]. Given the considerably high level of antimonial resistance [12] and the need to develop strategies to improve the treatment of patients [12], [13], a clinical score to predict SbV treatment failure would have an important impact on the control efforts for CL leishmaniasis.

This study evaluates potential risk factors and disease severity parameters for SbV treatment failure of ulcerative cutaneous leishmaniasis. A predictive score for antimonial treatment failure (PSATF) is generated in a nested case-control study conducted in Peru.

## Materials and Methods

### Patients

We performed a nested case-control study from a prospective cohort that took place between November 2001 and December 2004 at the Leishmaniasis Clinic of the Instituto de Medicina Tropical “Alexander von Humboldt,” Universidad Peruana Cayetano Heredia, in Lima, Peru. The clinic serves patients from nearly all areas of endemicity in the country.

Subjects from both sexes and all ages with a first episode of parasitologically confirmed CL by direct Giemsa stained smear or positive culture were recruited. Patients with only ulcerative lesions and with a first diagnosis of CL without mucosal, disseminated or diffuse lesions, who received at least 20 doses of antimonials, and who were followed for at least 6 months after starting SbV treatment were included.

The patient's population included in this study was a sub-cohort of a larger sample previously reported [8]. Written, informed consent was obtained from all patients. In case of children their parents or guardians gave consent. Research protocols complied with national and international ethics policies. The human experimentation guidelines of the Institute of Tropical Medicine Antwerp were followed. Ethics clearance was obtained from the ethical committees of the Universidad Peruana Cayetano Heredia and the Institute of Tropical Medicine Antwerp, Belgium. Clinical and epidemiological data for each patient were available from a previous study [8].

### Chemotherapy

Patients received treatment on site, with standard supervised daily administration of generic sodium stibogluconate (SSG) from Colombia (Viteco SA, lots: 10700, 10800, 20600, 20700 and 30600) or India (Albert David Ltd, lot: 3P12001) following Ministry of Health (MHO) of Peru guidelines (20 mg Sb5+/kg/day), for 20 days by intravenous or intramuscular injection. Quality control for SbV concentration in all batches was performed by the International Dispensary Association (Amsterdam, The Netherlands). Follow-up visits were scheduled for 1, 2, 3, 6, and 12 months after treatment ended. Patients with treatment failure received either a repeat course of antimonials with or without topical imiquimod (Aldara; 3 M Pharmaceuticals) or intravenous amphotericin B (amphotericin B deoxycholate; Bristol-Myers Squibb).

### Treatment outcome

After completion of the therapy, patients were schedule at 1, 2, 3, 6 and 12 month to evaluate the progression of the disease. On each visit the patient was clinically classified as (1) cure, if complete wound healing, with epithelization and absence of any sign of activity or inflammation or (2) failure, if increased inflammation around the initial lesion, with or without epithelization, clinical reactivation of a healed lesion, or presence of new lesion(s) or a satellite lesion around the initial one was evidenced. Pending was record in patients that evidence lesion in progress to closure. Treatment failure was considered if lesion received clinical classification of failure after 3 months of follow up. Patients clinically classified as cure continued the follow up scheme in order to monitor relapse.

### Potential risk factors for treatment failure

Information available for patients included age, sex, body mass index (BMI), main occupation, geographical region where disease was acquired, and disease severity parameters. Main occupation was classified as low or high risk for exposure to insect bites. High-risk occupations included agriculture, mining, and logging. Lesion description included number, type, location, and lymph node compromise. Typing of the leishmania species isolated from patients was performed as previously described [8].

Parameters that reflect the severity of the ulcerative lesions were measured. These included time of the disease and lesion size, which was calculated as (transversal diameter/2)×(sagital diameter/2)×3.14. On patients with multiple ulcerated lesions, the total area was calculated considering areas of the three largest lesions. Time of disease was defined as the time period since the recognition of the first lesion until the start of treatment.

Another parameter considered was the presentation of “concomitant-distant” lesions at the time of enrollment. This parameter was defined as the appearance of more than one lesion in different segments of the body (head, arms, trunks and legs) within 15 days. Cutaneous lesions that appeared in the same body segment were considered satellital lesions but not “concomitant-distant”.

In all cases age was expressed in years, time of disease in days, and the total area of lesion in cm^2^. Gender was considered 0 if female and 1 for male. Genotype *L. braziliensis* was considered “1” if leishmania type is *L. braziliensis*, otherwise it was considered “0”. The covariate *low/high risk activity* is considered “1” in case of a high-risk occupation otherwise it was considered “0”. The concomitant-distant ulcers category was “1” if it was present, otherwise it was considered “0”.

### Statistical analysis

Categorical variables were described by frequencies and proportions. Continuous variables were described by means, medians and standard deviation. Categorical variables were compared by using chi-squared test. t-test and ANOVA were used to compare normally distributed variables while U Mann-Whitney and Kruskal-Wallis tests were used for non-normal distributed variables.

The probability of treatment failure was modeled in a multiple logistic regression. Potential clinical risk factors and severity indicators were tested as predictors after adjusting for potential confounders. Interactions and second order effects were tested. Multi-dimensional outliers were assessed with the test of Hadi [14]. Covariates that were significant in the univariate analysis and were not over-correlated with other covariates (correlation coefficient less than 0.75) were included in the multiple regression analysis. Nested models were compared with the likelihood ratio test. The best sensitivity and specificity of the PSATF was estimated by maximizing the Youden's index, J = sensitivity+specificity−1 [15].

A clinical tool to predict antimony treatment failure was created using the best multiple logistic regression model. The probability of treatment failure was calculated as:
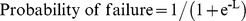
Where L is the linear predictor of the best multiple logistic model (a_0_+a_1_ X_1_+a_2_ X_2_+….+a_n_ X_n_, where X_i_ are the significant covariates included in the best multiple model, and a_i_ are the regression coefficients. All the statistical analyses were conducted with a 5% significance level using the software Stata 10.

## Results

A total of 171 patients received a diagnosis of leishmaniasis. In all these patients the parasite was isolated and typed during the study period. Of these patients, 87 met the eligibility criteria for ulcerative lesions and were included in the analysis. Eighteen patients (20.7%) failed the treatment and 69 (79.3%) cured. The age distribution ranged from 0.3–85 yr with a mean of 29 yr. Thirty patients (34.5%) were female and 57 (65.5%) were male. Fifty-two patients (59.8%) presented single lesions. The number of lesions in patients classified as multiple lesions ranges from 2 to 7. Among patients with multiples lesions, 10 (23%) showed “concomitant-distant” lesions.

A total of 84 patients did not meet eligibility criteria and were excluded from the analysis: 14 had previously received treatment for leishmaniasis, 18 presented mucosal involvement, 10 did not complete the first round of SbV treatment, 14 were followed-up for only 6 months, and 28 presented non-ulcerative lesions (nodule, plaque, mixed).

Descriptive statistics were compared between patients that cured and failed to treatment (Table 1). In the univariate analysis, patients who failed treatment were younger (mean = 16.2 years) than patients that cured (mean = 32.4 years) (P<0.001). Treatment failure was significantly associated with the type of work activity. People living in areas with a high rate of insect bites presented lower risk of chemotherapy failure (P<0.001). Time of disease and the size of induration, although border line significant, were included in the logistic regression analysis.

**Table 1:** Epidemiologial, clinical and laboratory characteristics of patients with ulcerative cutaneous leishmaniasis stratified by cure or failure condition with pentavalent antimonial treatment in Peru.

Variable	Cure	Failed	P-value
Gender			0.318
Male	22 (73.3%)	8 (23.67%)	
Female	47 (82.46%)	10 (17.54%)	
Age (mean+−SD)	32.36+−19.72	16.17+−12.81	0.001
Activity			0.001
Low risk	30 (65.22%)	16 (34.78%)	
High risk	38 (95%)	2 (5%)	
Geographic location			0.90
Central and northern coast	27 (81.82%)	6 (18.18%)	
High amazone	31 (79.49%)	8 (20.51%)	
Southern Andes	2 (66.67%)	1 (33.3%)	
Low Amazone	9 (75%)	3 (25%)	
Duration of disease^*^ (days)	77.5 (45–114)	60 (31–86)	0.068
Total area of lesion(s)^*^ (cm2)	2.71 (1.32–3.93)	1.27 (0.34–2.07)	0.007
Total number lesions^*^	1 (1–2)	2 (1–2)	0.95
Skin test diameter (mm) Mean+−SD	9.12+−2.92	7.41+−1.37	0.053
Concomitant-distant lesions			0.015
No	64 (83.12%)	13 (16.88%)	
Yes	5 (50%)	5 (50%)	
Genotype			0.123
*L. guyanensis*	22 (91.67%)	2 (8.33%)	
*L. braziliensis*	17 (68%)	8 (32%)	
*L. peruviana*	30 (78.95%)	8 (21.05%)	

Mean and standard deviation (SD) or frequencies were compared between cured and failed cases to antimony chemotherapy patients when normally distributed covariates were involved.

Median and (25^th^–75^th^) percentiles (inter-quartile range) were compared in variables with non normal distribution.

Among the clinical parameters that define the severity of the disease, the total area of lesions and the presence of “concomitant-distant” lesions, were significantly associated with treatment failure. Total area of lesions in patients that cured was greater than in patients that failed treatment (2.7 cm^2^ vs 1.26 cm^2^, U-Man-Whitney test, P = 0.007). Fifty percent of people with concomitant-distant lesions failed to antimony chemotherapy whereas only 17% failed among the patients that presented either single or multiple non-concomitant-distant lesions.

The unadjusted odds ratios estimated from the univariate analysis for treatment failures are shown in Table 2. Covariates significantly associated with treatment outcome were: Age, occupation, parasite species induration size, the natural logarithm of the total area of lesion, and the presentation of concomitant-distant lesions. The presence of concomitant-distant lesions showed a remarkably highly significant association in the univariate model (OR = 4.92, P = 0.023). The number of lesions was not significantly associated with treatment failure. The logarithmic transformation of the total area of lesion evidenced a significant association (OR = 0.50, P = 0.006).

**Table 2:** Logistic regression models to predict antimony treatment failure of ulcerative cutaneous leishmaniasis.

Factor	Univariate analysis^a^ OR (95% CI)	P-value	Multiple-variable analysis 1^b^ OR (95% CI)	P-value	Multiple-variable analysis 2^c^ OR (95% CI)	P-value
Age	0.94 (0.89–0.97)	0.003	0.88 (0.81–0.97)	0.010	0.92 (0.88–0.98)	0.008
Duration of disease	0.99 (0.97–1.00)	0.138	0.97 (0.95–0.99)	0.025	0.97(0.94–0.99)	0.02
high-risk/low risk Activity	0.098 (0.02–0.46)	0.003	0.07 (0.009–0.54)	0.011	0.12(0.02–0.75)	0.02
Number of lesion(s)	0.93 (0.32–2.7)	0.896				
Total area of lesion(s)*	0.499 (0.31–0.82)	0.006	0.53 (0.27–1.04)	0.065		
Concomitant-distant lesion(s)						
No	1		1		1	
Yes	4.92 (1.24–19.48)	0.023	30.5 (1.67–558.56)	0.021	6.27(0.96–40.7)	0.054
Leishmania species						
*L guyanensis*	1		1			
*L braziliensis*	5.17 (0.97–27.60)	0.054	25.7 (2.34–282.30)**	0.008		
*L peruviana*	2.93 (0.56–15.19)	0.200	1			

a Odds ratio and its 95% confidence interval of the simple logistic regression in the univariate analysis.

b Odds ratio and its 95% confidence interval of the multiple logistic regression for the Prognostic Score 1. R^2^ = 0.54; 85 patients in the model.

c Odds ratio and its 95% confidence interval of the multiple logistic regression for the Prognostic Score 2 (without inclusion of the Leishmania species). R^2^ = 0.38; 85 patients in the model.

*: This variable was log-transformed before the analysis.

**: Odds ratio obtained after pooling *L. guyanensis* and *L. peruviana* and comparing against *L. brasiliensis*.

The best multiple logistic model to explain treatment failure included six covariates significantly associated with treatment outcome (Table 2). The regression coefficients of the linear predictor of the best multiple logistic model were used to calculate the PSATF-1 (PS1 = 1/(1+e ^−L^_1_), where L_1_ = 6.617−0.12 (age)+3.24 (L. braziliensis)−0.027 (Time of disease)−0.64 (log total area lesion +1)+3.41 (Concomitant-distant)−2.65 (low/high risk activity)). Noteworthy, the presence of concomitant-distant lesions and the type of *Leishmania* species were strongly associated with treatment failure (odds ratios of 30.5 and 25.7 respectively). Treatment failure predictors associated with odds ratios ranging from 6 to 30, showed a statistical power from 0.64 to 0.99 respectively.

**Figure 1:**
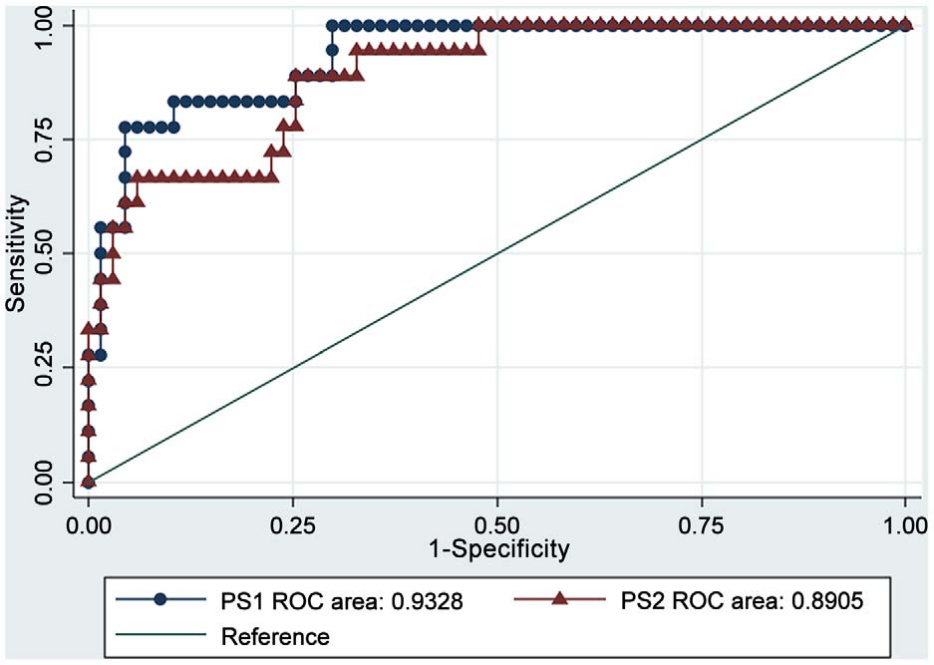
Receiver Operating Curve for the Prognostic Score 1 (PS1) and Prognostic Score 2 (PS2) of antimony chemotherapy treatment failure.

The best model explained 54% of the variability of treatment failure. The total area under the Receiver Operating Curve was 0.93 (Figure 1). The best sensitivity and specificity that maximized the Youden's index were 77.78 and 92.52 respectively, for a PS1 cutoff of 0.4 (if PS1>0.4 failure is prognosticated, otherwise it is expected a cure). Different sets of sensitivity and specificity of PS1 were tabulated for different cutoff values (Table 3).

**Table 3:** Sensitivity and specificity to predict antimony treatment failure for the different cutoffs of the linear scores for the Prognostic Scores 1 and 2.

	Prognostic Score 1		Prognostic Score 2	
Probability cutoff	Sensitivity	Specificity	Sensitivity	Specificity
0.6	55.56	98.51	44.44	98.51
0.5	72.22	95.52	61.11	95.52
0.4	77.78	95.52	66.67	92.54
0.37	77.78	94.03	66.67	89.55
0.35	77.78	94.03	66.67	85.07
0.3	77.78	91.04	66.67	83.58
0.29	77.78	91.04	66.67	82.09
0.27	83.33	88.06	66.67	77.61
0.25	83.33	86.57	77.78	76.12
0.23	83.33	85.07	77.78	76.12
0.2	83.33	76.12	77.78	74.63

Prognostic Score 1. Linear score model based on the multiple logistic regression that included the six significant covariates described in Table 2.

Prognostic Score 2. Linear score model based on the multiple logistic regression that included the four significant covariates described in Table 2.

In addition we evaluated a score to model the chemotherapy failure without considering the leishmania specie (PSATF-2 (PS2)). This multivariate model included four significant variables and was able to explain 38.7% of the chemotherapy failure (Figure 1). Concomitant-distant lesions remained the most important predictor of chemotherapy failure (OR = 6.27, although significance was 0.054). The optimal sensitivity and specificity of PS2 were determined by maximizing the Youden index and reached 66.67% and 92.54% respectively, which corresponded to a PS2 cutoff of 0.4.

The regression coefficients of the linear predictor of the reduced multiple logistic model (Table 4), were used to calculate the PSATF-2 (PS2 = 1/(1+e ^−L^_2_), where L_2_ = 2.84−0.073 (age)−0.028 (Time of disease)+1.83 (Concomitant-distant)−2.06 (low/high risk activity)). A free Excel-based electronic calculator of PSATF-1 and PSATF-2 (Calculator S1) is available at the online supplementary material or upon request to the authors.

**Table 4:** Regression coefficients of the logistic models for chemotherapy failure.

Variable	Prognostic Score 1	P-value	Prognostic Score 2	P-value
Age	−0.12	0.01	−.073	0.07
Duration of disease	−0.03	0.02	−0.03	0.02
Total area of lesión(s)	−0.64	0.06		
Concomitant-distant lesion(s)	3.41	0.02	1.83	0.05
Low/high risk activity	−2.65	0.01	−2.06	0.02
*L. brasilienzis*	3.25	0.01		
Constant term	6.62	<0.01	2.84	0.02

## Discussion

We report here for the first time a score for prognosis of antimonial therapeutic failure in ulcerative CL patients treated with SSG. This prognostic score PSATF (PS1) includes a newly identified risk factor that was highly associated with the risk of treatment failure: the appearance of concomitant-distant lesions. The proposed prognostic score could be used as a clinical prediction tool able to be adjusted (PS2 instead of PS1) according to the level of the technical capacity available on site.

The two most important factors included in the PSATF, were the appearance of concomitant-distant lesions and the species of Leishmania associated to infection. The appearance of concomitant-distant lesions compared to patients with unique or non-concomitant-distant multiple lesions appear as a promising important clinical finding to be considered during treatment of leishmaniasis. Although a relatively small number of cases give support to this finding, the high OR (30.5) is statistically significant (p = 0.023). However, a larger cohort study will be required to confirm this finding. Confirming the findings in other studies [8], [10], infection with *L. braziliensis* also accounts as an important risk factor for treatment failure (OR = 25.7).

A limitation is that concomitant-distant lesions may not be identified at a very early stage of the disease. However, considering that in developing countries, patients seek medical attention lately, this limitation would not prevent the appropriate use of the prognostic score in the majority of situations.

As showed in Table 1 Previous works by our group and others [10], [16] showed the relationship between the area of lesion and the chemotherapy failure. This suggests that contrary to what is a common concept of treatment of leishmaniasis, early treatment and in consequence a smaller lesion size seems to be a risk for chemotherapy failure.

The frequency of SbV treatment failure estimated in this study was as high as reported in other sites [8]–[10]. About 20% of patients fail to SbV treatment in a first treatment scheme. Therefore it is important to predict failure with a reasonable sensitivity and specificity. The PSATF proposed could provide different combinations of sensitivities and specificities, according to specific necessities. The PSATF cut off of 0.4 has privileged the specificity over sensitivity to optimize the safety and rational use of chemotherapy with SbVs. The optimal values of sensitivity and specificity indicate that 78% of patients who failed treatment were correctly predicted while 92.5% of patients who cured were classified as such. In this way a larger proportion of patients will be correctly treated with the drug that is provided free of costs by the ministry of health while avoiding the use of second line drugs that have adverse side effects and are more expensive [17], [18]. Similar approaches to determine prognostic scores of treatment failure have been proposed for other diseases such as tuberculosis [19]. Clinical scores were also developed to predict fatal outcome in patients with visceral leishmaniasis [20], [21].

Our proposed PSATF includes two different models depending on the availability of genotyping of *Leishmania* species. Given that in some settings genotyping is not possible to perform, the use of model PS2 that does not require genotyping appears to be an alternative to improve the management of this disease in those places. It is important to highlight that the sensitivity and specificity are not largely compromised when the species of leishmania is excluded.

The appearance of concomitant-distant lesions was highly associated with the risk of treatment failure in contrast to the presence of multiple lesions regardless the timing of onset. The total number of lesions has been previously suggested to be a risk factor for SbV treatment failure [9], [10], but in these studies, concomitant-distant lesions were not distinguished.

Cutaneous leishmaniasis is characterized by lesion(s) that progress from an erythema to the typical ulcerative form in a range of 2–24 weeks [22]. All the patients included in the study were clinically classified as ulcerative cutaneous leishmaniasis and the number of lesions range from 1 to 7. Concomitant distant lesions do not seem to be the typical clinical form of disseminated leishmaniasis since it is characterized by the presence of numerous ulcerative and papular lesions.

Given its remarkable importance, it is likely that the appearance of concomitant-distant lesions correspond to a different biological phenomenon that needs to be further studied. A possibility is that these lesions could be a consequence of an intrinsic immune failure that favors metastasis [23], or a consequence of multiple infected sand-fly bites on different parts of the body [24]. However, in both cases it might indicate a decreased capability of the immune system to undergo a cell-mediated immunity against leishmania parasites.

The PSATF here presented has practical advantages because it depends on observable clinical and epidemiological features, easily obtained by physicians or health workers. With the increased use of portable computational systems, the prognostic score PSATF could be easily used by physicians in tablet PCs and smartphones. Prospective clinical studies should probe its value as prognostic tool.

## Supporting Information

Calculator S1 **Excel-based calculator for PS1 and PS2.**
(XLS)
